# Screening of bacteriocin-producing dairy *Enterococcus* strains using low-cost culture media

**DOI:** 10.3389/fmicb.2023.1168835

**Published:** 2023-06-02

**Authors:** Inna L. Garmasheva, Ljubov T. Oleschenko

**Affiliations:** Department of Physiology of Industrial Microorganisms, Danylo Zabolotny Institute of Microbiology and Virology, National Academy of Science of Ukraine, Kyiv, Ukraine

**Keywords:** *Enterococcus*, bacteriocin, *Listeria monocytogenes*, screening, low-cost media

## Abstract

This study was carried out to select the bacteriocinogenic strains among *Enterococcus* strains isolated from Ukrainian traditional dairy products using a low-cost media for screening, that containing molasses and steep corn liquor. A total of 475 *Enterococcus* spp. strains were screened for antagonistic activity against *Pseudomonas aeruginosa*, *Escherichia coli*, *Staphylococcus aureus*, and *Listeria monocytogenes* indicator strains. The initial screening revealed that 34 *Enterococcus* strains during growth in low-cost medium containing corn steep liquor, peptone, yeast extract, and sucrose produced metabolites with inhibition activity against at least of the indicator strains used. Enterocin genes *entA*, *entP*, and *entB* were detected in 5 *Enterococcus* strains by PCR assay. Genes of enterocins A and P were found in *E. faecalis* 58 and *Enterococcus* sp. 226 strains, enterocins B and P – in *Enterococcus* sp. 423, enterocin A – in *E. faecalis* 888 and *E. durans* 248 strains. Bacteriocin-like inhibitory substances (BLIS) produced by these *Enterococcus* strains were thermostable and sensitive to proteolytic enzymes. To our knowledge, this is the first report on the isolation of enterocin-producing wild *Enterococcus* strains from traditional Ukrainian dairy products using a low-cost media for screening bacteriocinogenic strains. Strains *E. faecalis* 58, *Enterococcus* sp. 423, and *Enterococcus* sp. 226 are promising candidates for practical use as producers of bacteriocins with inhibitory activity against *L. monocytogenes* using molasses and steep corn liquor as cheap sources of carbon and nitrogen, that can significantly reduce the cost of industrial bacteriocin production. Further studies will be required to determine the dynamic of bacteriocin production, its structure, and mechanisms of antibacterial action.

## 1. Introduction

Enterococci are widely distributed in nature and one of the main niches of their habitat are fermented milk products obtained by spontaneous fermentation of raw milk. Natural resistance to a wide range of temperatures, pH, and high NaCl concentrations allows them to survive in cheeses but they are also commonly found in other fermented milk products, for example artisanal fermented milks such as Kumis and Dahi (Giraffa, 2003; Ogier and Serror, 2008; Chaves-López et al., 2011; Farah et al., 2016). Due to their enzymatic activities, *Enterococcus* strains can impart special organoleptic characteristics to cheeses and are often included in starter cultures. Enterococci can also improve the microbiological quality and extend the shelf life of end-products due to their antagonistic properties against pathogens (Giraffa, 2003; Foulquié Moreno et al., 2006). According to the literature, one of the mechanisms of the antagonistic action of enterococci is their ability to produce bacteriocins, which allows the use of bacteriocinogenic *Enterococcus* strains as protective starter cultures, as well as producers of bacteriocins that can be used as purified preparations (Ng et al., 2020). Bacteriocins of lactic acid bacteria (LAB) have been intensively studied over the past decades and there is still more interest in screening for new producer strains (Nami et al., 2019; Lauková et al., 2020; Choeisoongnern et al., 2021; Sosa et al., 2021). According to a definition, bacteriocins are ribosomally synthesized peptides that inhibit the growth of phylogenetically closely related microorganisms (Darbandi et al., 2022). As was shown in numerous studies, LAB bacteriocins can also inhibit the growth of many opportunistic and foodborne pathogens, and are of great practical interest as natural preservatives in the food industry (Sameli et al., 2021; Kasimin et al., 2022). Bacteriocins of enterococci, so-called enterocins, mainly belong to subclass IIa, which are small, heat-stable peptides with antagonistic activity against the *Listeria monocytogenes* and other pathogenic bacteria (Darbandi et al., 2022; Kasimin et al., 2022). Also, anticancer, antiviral, and antibiofilm activities of enterocins were reported (Ermolenko et al., 2019; Lee et al., 2021; Sharma et al., 2021). Such a wide range of biological activity of enterocins significantly expands the potential field of the practical application of *Enterococcus* strains, including as probiotics (Nami et al., 2019; Ng et al., 2020; Choeisoongnern et al., 2021).

The composition and the concentration of nutrients in the medium greatly influence LAB bacteriocin production (López et al., 2007). Most studies on the production of enterocins have been conducted using commercial laboratory media such as MRS, BHI, and M17. Commonly, after the initial screening of bacteriocinogenic LAB strains, the next stage is the optimization of culture conditions for large-scale biomass or bacteriocin production using cheaper cultural media (Metsoviti et al., 2011; Schirru et al., 2014; Sosa et al., 2021). Data concerning enterocin production by *Enterococcus* strains using inexpensive media are very rare and mainly concern certain strains (Coetzee et al., 2007; López et al., 2007; Bali et al., 2014).

However, there is evidence that strains selected as bacteriocinogenic in the MRS medium do not produce bacteriocin when grown on media based on cheaper substrates, since the synthesis of bacteriocins strongly depends on growth conditions (Audisio et al., 2001; Todorov and Dicks, 2005; Schirru et al., 2014; Vandera et al., 2019). Thus, it is more reasonable to initially carry out primary screening on cheap media suitable for industrial production.

The aim of this study was the screening of potentially bacteriocinogenic *Enterococcus* strains, using low-cost by-product-based media among dairy-derived *Enterococcus* strains. To the best of our knowledge, this is the first report about the presence of bactericonogenic strains among enterococci isolated from artisan Ukrainian traditional dairy products prepared by spontaneous fermentation.

## 2. Materials and methods

### 2.1. Bacterial strains

The 475 *Enterococcus* strains from the Lactic Acid Bacteria Culture Collection of the Department of Physiology of Industrial Microorganisms, Zabolotny Institute of Microbiology and Virology NAS, (Kyiv, Ukraine) were used in this study. The *E. faecalis* (*n* = 350), *E. durans* (*n* = 92), *E. faecium* (*n* = 15). *Enterococcus* spp. (*n* = 17) and 1 *E. hirae* strains were previously isolated from Ukrainian traditional dairy products such as fermented milk, cottage cheese, bryndza, and sour cream (Garmasheva et al., 2018). The strains were stored in MRS broth (HiMedia, India) supplemented with 30% glycerol at −50°C. *Enterococcus* strains were subcultured in MRS broth at 37°C for 24 h. The cultures were activated by two successive transfers in the MRS broth before use.

### 2.2. Initial screening for antagonistic activity

Production of antimicrobial metabolites was tested using four media having the following composition (w/v): medium I – 1.00% peptone, 0.05% yeast extract, 0.25% corn steep liquor, 2.00% sucrose; medium II – 1.00% peptone, 0.05% yeast extract, 0.25% corn steep liquor, 2.00% sugar beet molasses; medium III – 1.00% peptone, 0.05% yeast extract, 0.25% corn steep liquor; medium IV – 2.00% sugar beet molasses (pH 7.0 ± 0.2). Corn steep liquor and sugar-beet molasses were procured from local companies Interstarch Ukraine (Kyiv, Ukraine) and Radekhivskyi Sugar LLC (Ternopil region, Ukraine), respectively. The MRS broth also was used. All media were sterilized by autoclaving at 121°C for 15 min. Samples of 24 h-old *Enterococcus* cultures were serially diluted in sterile saline solution, plated on MRS agar and viable cell counts were determined for each medium used.

The antagonistic activity was determined by the well-diffusion assay as described previously (Schillinger and Lücke, 1989). The 6 mm-diameter stainless steel cylinders were laid on Petri dishes with Nutrient agar (NA), and the dishes were overlaid with 15 mL of molten NA (0.75%) inoculated with 100 μL of an overnight culture of the indicator strain. The *Pseudomonas aeruginosa* АТСС 9027, *Escherichia coli* ATCC 25922, *Staphylococcus aureus* ATCC 25923, and *Listeria monocytogenes* NCTC 5105 were used as indicator strains. After solidification, the cylinders were removed, and aliquots of 24 h-old *Enterococcus* cultures (50 μL) were poured into wells and kept at 4°C for 2 h. Then the Petri dishes were incubated for 24 h at 37°C. A clear zone around the wells was accepted as a positive result. Each determination was carried out in duplicates.

### 2.3. Screening of the enterocin-encoding genes by PCR

The total DNA was extracted from overnight cultures as described previously (Yost and Nattress, 2000). Briefly, 1.5 mL of overnight culture was centrifugated at 5000 rpm for 5 min, and the cell pellet was resuspended in 1 mL of sterile distilled water and centrifugated at the same condition. The resulting bacterial pellet was resuspended in 50 μL of sterile distilled water, heated at 95°C for 5 min, and centrifuged at 13000 rpm for 15 min. Then the supernatant was used as a template DNA in the PCR reaction. To investigate the occurrence of enterocin structural genes among *Enterococcus* strains, we performed a screening using primers specific to three well-known enterocin genes: for enterocin A – the forward primer 5´-GGTACCACTCATAGTGGAAA-3′ and reverse primer 5´-CCCTGGAATTGCTCCACCTAA-3′; for enterocin B – the forward primer 5´-СAAAATGTAAAAGAATTAAGATCG-3′ and reverse primer 5´-AGAGTATACATTTGCTAACCC-3′; for enterocin P – the forward primer 5´-GCTACGCGTTCATATGGTAAT-3′ and reverse primer 5´-TCCTGCAATATTCTCTTTAGC-3′. PCR amplification conditions were as follows: initial denaturation for 5 min at 95°C, 30 cycles of denaturation for 30 s at 95°C, annealing at 58°C (for the primers of *entA*) or 56°C (for the primers of *entB* and *entP*) for 30 s and extension at 72°C for 30 s, and a final elongation step of 5 min at 72°C (De Vuyst et al., 2003). The amplified PRC products were examined using 1.5% (w/v) agarose gel in a TBE buffer containing ethidium bromide with a DNA ladder GeneRuler 100 bp (Fermentas, United States). Fragments with the specific expected sizes were recorded as positive results for each bacteriocin-encoding gene for each *Enterococcus* strain. Strain *E. durans* 140D UCM B-2530 (UCM – Ukrainian Collection of Microorganisms) harboring *entA*, *entB*, and *entP* genes (GenBank accession numbers OP740237, OP740236, and OP690158, respectively) was used as a positive control.

### 2.4. Effect of enzyme and heat treatment on antagonistic activity of cell-free supernatants

Cell-free culture supernatants (CFS) were obtained by centrifugation of 250 mL of the overnight cultures at 5,000 rpm for 10 min and sterilized through 0.45 μm pore-size filters. Antagonistic activity of CFS was determined using *L. monocytogenes* and *Enterococcus* spp. strains as indicators. The inhibition activity of CFS was expressed in arbitrary units per milliliter (AU/mL), which was defined as the highest reciprocal dilution of CFS that gave inhibition of indicator strains. For exclusion of antibacterial action of organic acids, CFSs were adjusted to pH 6.5 ± 0.1 using 1 N NaOH and sterilized. The neutralized and filtered supernatant was assayed for antagonistic activity against *L. monocytogenes* and *E. faecalis* 888 strains by agar well diffusion assay.

CFSs were treated with the α-amylase, lipase, α-chymotrypsin, proteinase K, pepsin, and trypsin, each at a final concentration of 1 mg/mL. After 1 h of incubation at 37°C, enzymes were inhibited by heating at 100°C for 5 min. Antimicrobial activity against *L. monocytogenes* and *E. faecalis* 888 was examined using the agar well diffusion assay. The untreated cell-free supernatants were used as controls. The CFSs were heated at 100°C for 10 and 20 min and in an autoclave at 121°C, for 20 min, and the antagonistic activity was determined. Unheated samples were used as controls. Each determination was carried out in duplicates.

### 2.5. Partial purification of BLIS

*Enterococcus* strains were grown in Medium I at 37°C for 24 h. The bacterial culture was centrifuged at 8000 rpm for 15 min and the cell pellet was discarded. The supernatant was adjusted to pH 6.5 and filtered through a 0.45 μm filter. Solid ammonium sulfate was gently added to the supernatant (to a final saturation of 20, 40, 60, 80%), stirred, and held overnight at 4°C. Precipitates were collected by centrifugation at 8000 rpm for 30 min at 4°C and resuspended in 2 mL of 20 mmol sodium phosphate buffer (pH 7.0). The inhibitory activity of protein precipitates against *E. faecalis* 888 and *L. monocytogenes* was evaluated. Each determination was carried out in triplicates. The dissolved partially purified bacteriocins were desalted and freeze dried for further investigation.

### 2.6. Statistical analysis

Cluster analysis, a one-way analysis of variance (ANOVA), multiple mean comparisons using LSD test, and graphing procedures were carried out with the Statistika 7.0 software (Systat Inc., United States). The correlation between antagonistic action and viable cell count during growth in different media was analyzed by using Pearson’s correlation coefficient tests. All data are presented as the mean ± standard deviation of means. Statistical significance was defined as *p* < 0.05.

## 3. Results

### 3.1. Initial antagonistic activity screening

Using the agar well diffusion assay the antagonistic activity of 475 *Enterococcus* strains against *P. aeruginosa*, *E. coli*, *S. aureus*, and *L. monocytogenes* strains was determined. The presence of a clear zone around the well was assessed as a positive result. The medium I with 2% sucrose was used for initial screening. Only 34 (7%) *Enterococcus* strains produced metabolites with antagonistic action against at least one indicator strain used. Thus, these *Enterococcu*s strains and 3 strains that had no antagonistic activity were selected for further investigation. Among them, 21 *Enterococcus* strains inhibited the growth of *S. aureus*, 13 strains – the growth of *P. aeruginosa*, 9 *Enterococcus* strains showed activity against *E. coli*, and only 3 strains – against *L. monocytogenes*. It should be noted, that zones of growth inhibition were larger for the *L. monocytogenes* strain (16–18 mm) compared to other indicator strains (8–10 mm). The antibacterial action against *S. aureus* and *E. coli* strains exhibited during growth in the medium I was lost after replacement of sucrose with molasses (Medium II) or their exclusion from the medium (Medium III). The strains *E. faecalis* 266 and *E. durans* 300a remained active against *P. aeruginosa*, and three strains – against *L. monocytogenes*. At the same time, 5 strains remained active against *E. coli*, 3 strains – against *S. aureus*, and 4 – against *P. aeruginosa* when grown in 2% molasses. We also used the MRS medium because MRS is the most commonly used medium for LAB antagonistic activity detection. All 37 *Enterococcus* strains, selected during the initial screening, showed antibacterial action against *P. aeruginosa*, 14 – against *E. coli*, 5 – against *L. monocytogenes*, and none – against *S. aureus*. In addition, when grown in media II, III IV, and MRS broth some strains showed antibacterial action that was not observed during the initial screening.

**Figure 1:**
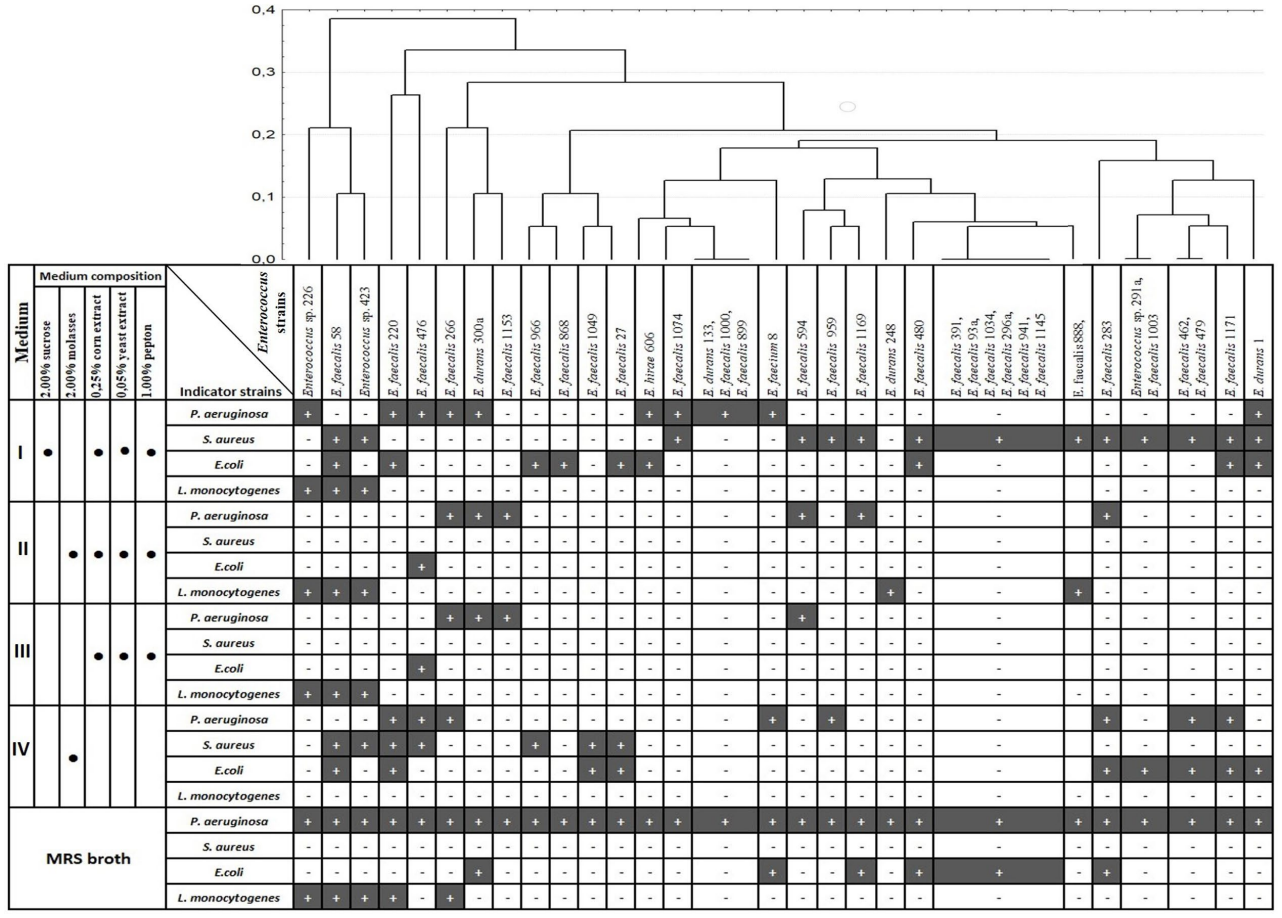
Dendrogram of antagonistic activity spectra of 37 *Enterococcus* strains depending on growth medium. Clustering was performed with Percent disagreement and UPGMA. Black bar (+) indicates the growth inhibition of indicator strain; medium component is included (•).

Cluster analysis was applied for analysis of the antagonistic activity spectra of the selected *Enterococcus* strains during growth on different media (Figure 1). Analysis of antibacterial activity in five media used revealed, that some strains had the same spectra of growth inhibition during growth in different media. Four *Enterococcus* strains showed only anti-*P. aeruginosa* activity.

**Figure 2:**
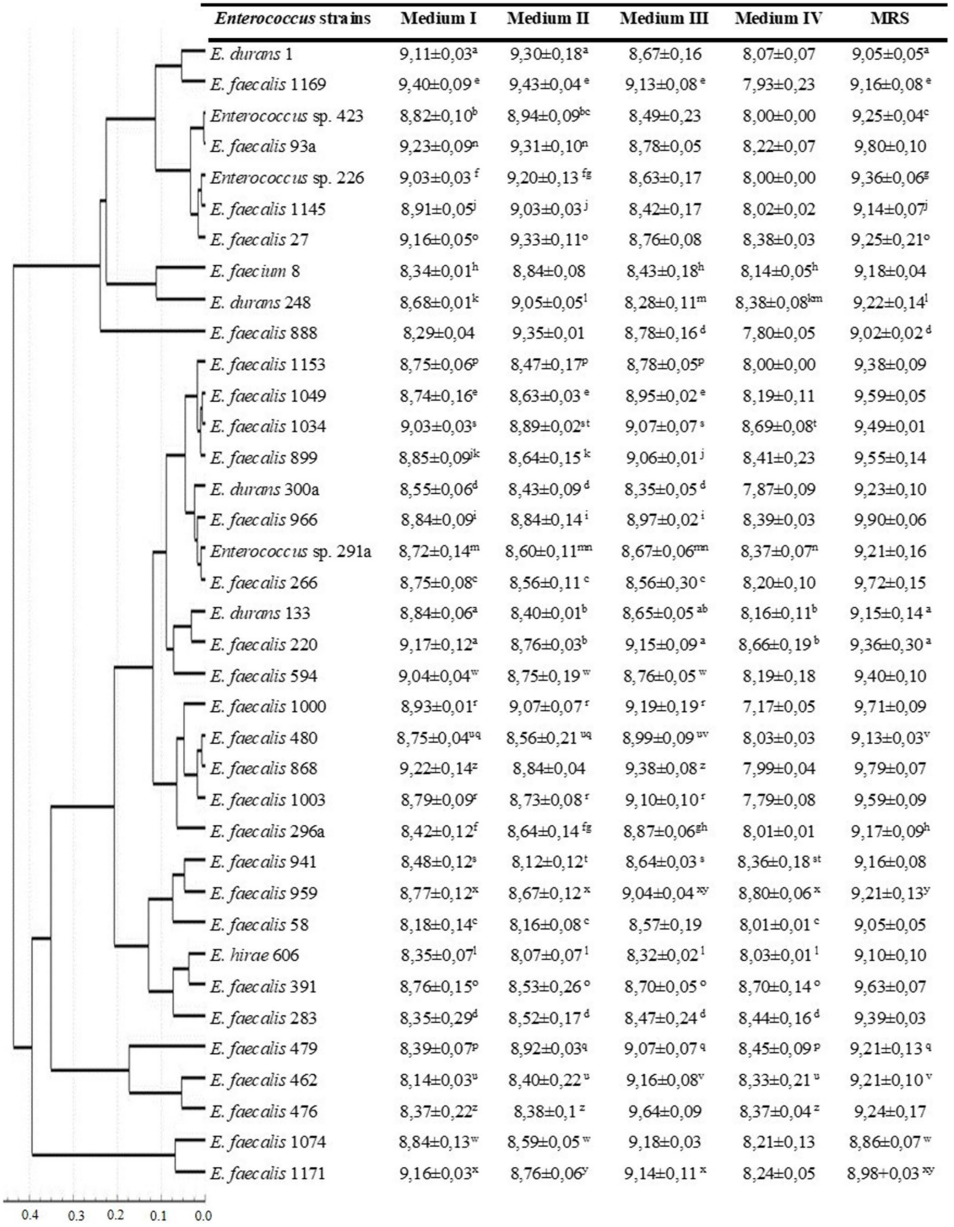
Viable cell counts of *Enterococcus* strains during growth in different media after 24 h of cultivation. Dendrogram based on viable cell counts (CFU ml^−1^) of *Enterococcus* strains after 24 h of cultivation in different media. Clustering was performed with Statistika 7.0, Pearson’s correlation coefficient and UPGMA. Mean values with different superscript letters within each row are significantly different (*p* < 0.05).

The viable cell count for the five media used is presented in Figure 2. The majority of the *Enterococcus* strains (54%) had higher viable cell counts in the MRS medium, and 62% of the strains had the lowest number of CFU ml^−1^ in 2% molasses (medium IV). Correlation analysis between antagonistic action and viable cell counts (CFU/ml) during growth in different media revealed that only one weak positive correlation (*r* = 0.34) was observed between CFU/ml value and ability to inhibit *P. aeruginosa* during growth on 2% molasses (Medium IV) (Table 1). A strong positive correlation was observed between anti-listerial activity during growth on different media (*r* = 0.54–1.00). Activities against *P. aeruginosa* and *E. coli* correlated between Medium II and Medium III (*r* = 0.79 and 0.70 respectively). Antagonistic action against *S. aureus* during growth in 2% molasses (Medium IV) weakly correlated (*r* = 0.3) with antagonistic action against *E. coli* during growth on Media I and II and *L. monocytogenes* during growth on Medium II and III (Table 1).

**Table 1:** Correlation coefficients between viable cell count of *Enterococcus* strains (CFU ml^−1^) and ability of enterococci to inhibit growth of indicator strains depending on growth medium.

Inhibited indicator strain	Medium I					Medium II				Medium III				Medium IV				MRS		
	*P. aeruginosa*	*E. coli*	*S. aureus*	*L. monocytogenes*	CFU ml^−1^	*P. aeruginosa*	*E. coli*	*L. monocytogenes*	CFU ml^−1^	*P. aeruginosa*	*E. coli*	*L. monocytogenes*	CFU ml^−1^	*P. aeruginosa*	*E. coli*	*S. aureus*	CFU ml^−1^	*E. coli*	*L. monocytogenes*	CFU ml^−1^
*Medium I P. aeruginosa 1*																				
*E. coli*	0.00	1																		
*S. aureus*	−0.55*	−0.13	1																	
*L. monocytogenes*	0.01	0.06	0.06	1																
CFU ml^−1^	−0.00	0.22	−0.07	−0.08	1															
*Medium II*																				
*P. aeruginosa*	−0.00	−0.26	−0.05	0.14	0.07	1														
*E. coli*	0.24	−0.10	−0.19	−0.05	−0.20	−0.08	1													
*L. monocytogenes*	−0.10	−0.04	0.03	0.75*	−0.20	−0.19	−0.07	1												
CFU ml^−1^	−0.13	−0.03	0.08	0.02	0.58*	−0.08	−0.18	0.22	1											
*Medium III*																				
*P. aeruginosa*	0.13	−0.20	−0.22	−0.10	0.01	0.79*	−0.06	−0.14	−0.20	1										
*E. coli*	0.09	−0.14	−0.27	−0.07	−0.15	0.22	0.70*	−0.09	−0.23	0.30	1									
*L. monocytogenes*	0.01	0.06	0.06	1.00*	−0.08	0.14	−0.05	0.75*	0.02	−0.10	−0.07	1								
CFU ml^−1^	−0.02	0.10	0.02	−0.25	0.24	−0.21	0.43*	−0.35*	0.07	−0.24	0.29	−0.25	1							
*Medium IV*																				
*P. aeruginosa*	0.13	−0.00	−0.06	−0.16	−0.29	0.12	0.32	−0.21	−0.17	0.02	0.16	−0.16	0.21	1						
*E. coli*	−0.21	0.31	−0.27	0.02	−0.09	−0.13	−0.11	−0.08	−0.03	−0.23	−0.16	0.02	0.12	0.37*	1					
*S. aureus*	−0.05	0.36*	0.23	0.36*	−0.01	−0.22	0.34*	0.21	−0.04	−0.17	0.19	0.36*	0.17	0.08	0.28	1				
CFU ml^−1^	−0.16	0.06	0.08	−0.19	−0.00	−0.11	0.12	−0.20	−0.18	−0.13	0.01	−0.19	0.03	0.34*	0.21	0.19	1			
*Medium MRS*																				
*E. coli*	−0.32	−0.33	0.25	−0.23	−0.12	0.25	−0.13	0.02	0.11	0.08	0.06	−0.23	−0.34*	−0.15	−0.41*	−0.39*	−0.01	1		
*L. monocytogenes*	0.23	0.14	−0.13	0.75`*	0.04	0.01	−0.07	0.54*	−0.03	0.12	−0.09	0.75*	−0.19	0.18	0.09	0.41*	−0.02	−0.31	1	
CFU ml^−1^	−0.09	−0.07	−0.34*	−0.12	0.31	0.10	−0.05	−0.23	0.12	0.16	−0.01	−0.12	0.20	−0.07	−0.15	0.11	0.01	0.04	−0.10	1

*Bold type and asterisks highlight the statistically significant correlation (*p* < 0.05).

### 3.2. Detection of enterocin genes

Among 37 tested, enterocin genes were observed in 5 *Enterococcus* strains (Table 2). The structural genes of enterocins A and P were found in *E. faecalis* 58 and *Enterococcus* sp. 226 strains, enterocins B and P – in *Enterococcus* sp. 423, and enterocin A – in *E. faecalis* 888 and *E. durans* 248 strains.

**Table 2:** PCR-detected structural enterocin gene profiles of *Enterococcus* strains.

Strain	Enterocin gene		
	*entA*	*entB*	*entP*
*E. faecalis* 58	+	−	+
*Enterococcus* sp. 226	+	−	+
*Enterococcus* sp. 423	−	+	+
*E. durans* 248	+	−	−
*E. faecalis* 888	+	−	−

“+”, strain possess enterocin gene; “−“, enterocin gene was absent.

### 3.3. Antagonistic activity against *Enterococcus* spp. strains

Each of the five *Enterococcus* strains harboring *entA*, *entB*, or *entP* genes was tested for antagonistic activity against 37 *Enterococcus* strains, that were selected after initial screening. *E. faecalis* 58, *Enterococcus* sp. 226, and *Enterococcus* sp. 423 showed antibacterial action against 27 *E. faecalis* strains (including *E. faecalis* 888 with *entA* gene detected by PCR) and 1 *E. hirae* strain. Four *E. durans*, 1 *E. faecium*, and 1 *Enterococcus* sp. strains were not sensitive. Furthermore, *E. faecalis* 58, *Enterococcus* sp. 226 strains, and *Enterococcus* sp. 423 did not inhibit the growth of each other and were resistant to their antimicrobial metabolites. These three strains were selected for further investigation of the nature of their antibacterial metabolites.

### 3.4. Antagonistic activity of cell-free supernatants

Neutralized cell-free supernatants (CFS) of *E. faecalis* 58, *Enterococcus* sp. 226, and *Enterococcus* sp. 423 strains expressed no inhibition of the growth of *P. aeruginosa*, *S. aureus*, or *E. coli* strains, but showed antagonistic activity (the same as overnight cultures with bacterial cells) against *L. monocytogenes* and *E. faecalis* 888 strains, that also was chosen as the indicator strain. Antagonistic activity of CFSs was observed to be at 51200 AU/mL against *L. monocytogenes strain* and 1,600–3,200 AU/mL against *E. faecalis* 888 strain.

### 3.5. Nature of bacteriocin-like substances

The antibacterial metabolites produced by *E. faecalis* 58 *Enterococcus* sp. 226 and *Enterococcus* sp. 423 strains were found to be heat stable as they could resist heat treatment at 100°C for 20 min and at 121°C for 20 min, although the inhibitory activity of the cell-free supernatants was lower after autoclaving (Table 3). The inhibitory activity was completely lost after treatments of the cell-free supernatants with proteolytic enzymes proteinase K, trypsin, pepsin, and pancreatin. The activity was not affected by treatment with α-amylase and lipase.

**Table 3:** Effect of neutralization, heat and enzyme treatments on inhibitory activity of cell-free supernatants, obtained after growth of *Enterococcus* strains in Medium I, against *E. faecalis* 888 and *L. monocytogenes.*

	Zone of inhibition (mm)					
	*E. faecalis* 58		*Enterococcus* sp. 423		*Enterococcus* sp. 226	
	*L. monocytogenes*	*E. faecalis* 888	*L. monocytogenes*	*E. faecalis* 888	*L. monocytogenes*	*E. faecalis* 888
CFS (control, pH 3.7 ± 0.1)	18.3 ± 0.3	17.0 ± 0.0	18.7 ± 0.3	15.3 ± 0.3	18.3 ± 0.7	16.7 ± 0.3
Neutralized CFS (pH 6.5 ± 0.1)	17.7 ± 0.7	16.3 ± 0.3	18.3 ± 0.3	15.0 ± 0.0	17.7 ± 0.3	15.7 ± 0.3
100° C for 10 min	17.7 ± 0.7	16.7 ± 0.3	18.0 ± 0.6	15.7 ± 0.3	18.0 ± 0.6	15.3 ± 0.3
100° C for 20 min	18.0 ± 0.6	16.3 ± 0.3	18.0 ± 0.6	16.0 ± 0.6	17.7 ± 0.3	15.7 ± 0.3
121° C for 15 min	13.3 ± 0.3	11.7 ± 0.3	13.3 ± 0.7	12.3 ± 0.3	13.7 ± 0.3	11.7 ± 0.3
Proteinase K	0	0	0	0	0	0
Trypsin	0	0	0	0	0	0
α-chymotrypsin	0	0	0	0	0	0
Pepsin	0	0	0	0	0	0
α-amylase	18.0 ± 0.6	15.7 ± 0.3	18.0 ± 0.6	15.3 ± 0.3	17.3 ± 0.3	15.7 ± 0.3
Lipase	17.3 ± 0.3	16.0 ± 0.6	18.0 ± 0.6	15.7 ± 0.3	18.0 ± 0.6	15.3 ± 0.3

### 3.6. Partial purification of BLIS

**Figure 3:**
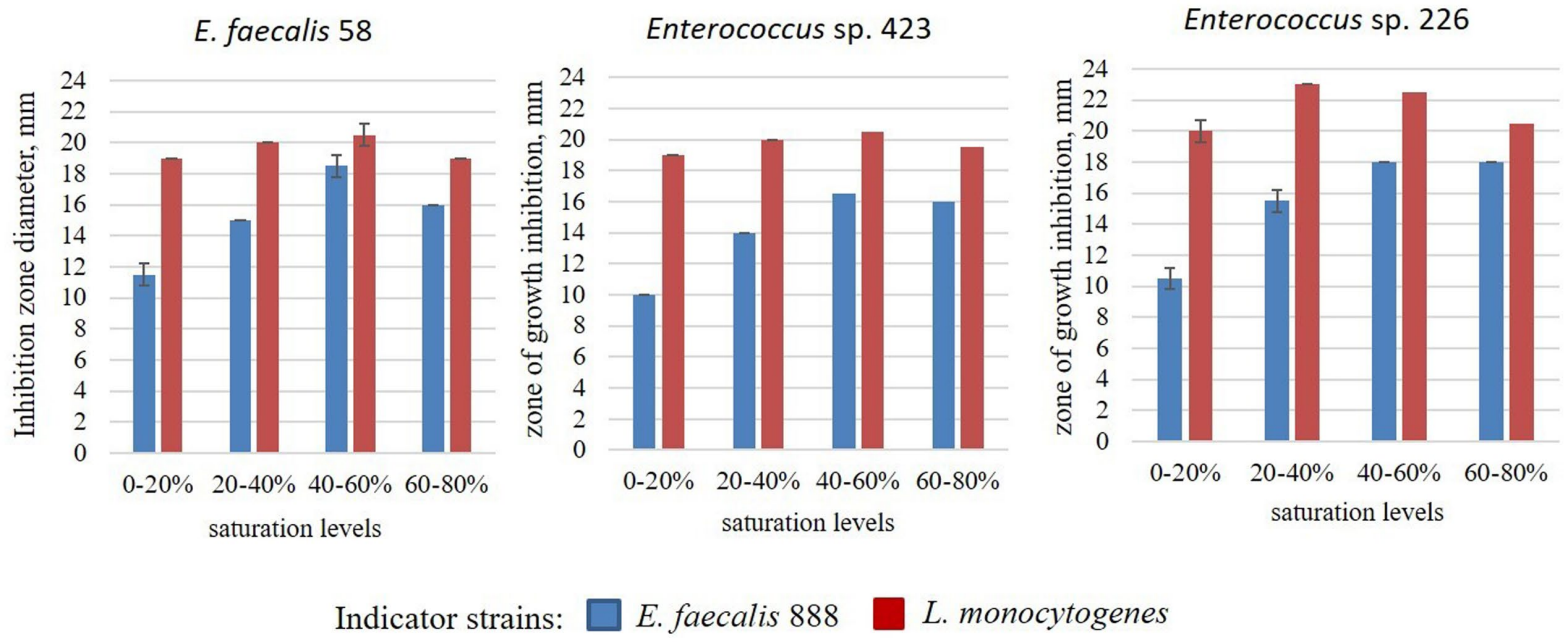
Antagonistic action of semi-purified BLIS of *Enterococcus* strains.

The size of the inhibition zones of the crude extracts of enterocins obtained by ammonium sulfate precipitation is presented in Figure 3. The antagonistic action of the crude extracts against *E. faecalis* 888 increased with increasing of ammonium sulfate concentration to 40–60%. At the same time, antagonistic action against *L. monocytogenes* was stronger compared to *E. faecalis* 888, it reached the maximum at 20–40% ammonium sulfate and did not change dramatically up to 80%.

## 4. Discussion

In this work, screening of potentially bacteriocinogenic strains among dairy-derived enterococci was carried out. At the initial screening stage, we evaluated the antagonistic activity of bacterial cultures that were grown in a medium containing sucrose, CSL, peptone, and yeast extract. Only 34 (from 475) *Enterococcus* strains were selected because they exhibited antagonistic action against at least one of the indicator strains. To our knowledge, this is the first report about using a low-cost medium for screening of bacteriocinogenic enterococci, because commonly MRS or other commercial media are used for initial screening. As was shown in many studies, MRS broth is the optimal medium for bacteriocin production by *Enterococcus* strains compared to other complex media, such as BHI broth or M17 broth (De Kwaadsteniet et al., 2005; Yang et al., 2018). But commercial growth media are too costly for industrial production. We can assume that the use of cheap media suitable for large-scale cultivation instead of MRS for screening will make it possible to avoid the stage of medium composition optimization to reduce the cost of bacteriocin production. In our work, we use four media containing sucrose, molasses, and CSL in different combinations. All these components were used for bacteriocin production by different *Enterococcus* strains and other LAB (Coetzee et al., 2007; Bali et al., 2014). The main carbohydrate in molasses is sucrose but its concentration may vary (Audisio et al., 2001), so for initial screening sucrose was used as a carbon source (Medium I). As was shown, sucrose is one of the well-preferred carbon sources for Nisin production by *L. lactis* strains (Parente and Ricciardi, 1999). Yeast extract and peptone also were added to the media, because these components are required for bacteriocin production, as previously shown (Mirhosseini and Emtiazi, 2011; Bali et al., 2014).

During growth in media containing sucrose or molasses as the carbon source, most enterococci strains had compatible viable cell counts. The absence in the culture medium of the main source of carbohydrates (sucrose or molasses) did not affect the growth of 75% of strains. During growth in 2% molasses, only 40,5% of strains had the same viable cell counts as in at least one of the other media used, with exception of MRS. Overall, the growth rate did not correlate with the growth inhibition of indicator strains.

Detection of genes encoding bacteriocin production by PCR is a commonly used method for screening of bacteriocin-producing LAB strains. The analysis of literature data showed that the presence of enterocin genes *entA*, *entB*, *entP*, *entL50A*, *entL50B*, *entAS-48*, *cyl*, or *bac31* is most often determined in enterococci by PCR analysis (Vandera et al., 2018, 2019; Furlaneto-Maia et al., 2020; Lauková et al., 2020a). According to the results obtained by the authors, genes of enterocin A, B, and P are most often detected in enterococci isolated from different econiches (Ogaki et al., 2016; Vandera et al., 2018; Kubašová et al., 2020). None of the *Enterococcus* strains analyzed by Vandera et al. (2018) possessed *entL50A*, *entL50B*, *entAS-48*, or *bac31* genes. Only the gene of enterocin P was detected in strain *E. lactis* Q1 using PCR amplifications with primers specific for genes *entA*, *entB*, *entP*, *LB50A*, and *LB50B* (Braïek et al., 2017). The structural enterocin genes entA, entB, entP, and entX were the most frequently observed in *Enterococcus* spp. isolated from food and environment, but genes encoding for enterocin L50A/B, Q, K, 31, and AS48 were not found (Furlaneto-Maia et al., 2020). Thus, in the present study, we focused on screening the most frequently occurring enterocin genes *entA*, *entB*, and *entP*. Enterocin typing by specific PCR revealed that only 5 from 37 antagonistic *Enterococcus* strains harbored the most widespread enterocin structural genes *entA*, *ent B*, and *entP*. According to data of literature, enterocins A (Aymerich et al., 1996), B (Casaus et al., 1997), and P (Cintas et al., 1997) are produced by *Enterococcus* strains isolated from different sources (Cocolin et al., 2007; Ogaki et al., 2016; Vandera et al., 2018; Furlaneto-Maia et al., 2020). Enterocins A, B, and P had a broad and very similar inhibitory spectrum against Gram-positive bacteria, such as *Listeria* spp., *Clostridium* spp., *Enterococcus* spp., and *Staphylococcus aureus*, but did not inhibit the growth of Gram-negative indicator strains (Aymerich et al., 1996; Casaus et al., 1997; Cintas et al., 1997). Multiple-enterocin-producing enterococci are also frequently found in raw milk and artisanal dairy products (Furlaneto-Maia et al., 2020). Strains *Enterococcus* sp. 423, 226, and *E. faecalis* 58 harbor a combination of two enterocin genes (*entB*/*entP* and *entA*/*entP*, respectively), and exhibit anti-listerial and anti-enterococcal activity, CFSs of these stains retained antagonistic activity after neutralization. Thus, the activity spectra of *E. faecalis* 58, *Enterococcus* sp. 226 and 423 correspond to the data previously presented by other authors regarding the activity of enterocins A, B, and P (Aymerich et al., 1996; Casaus et al., 1997; Cintas et al., 1997). But, neutralized CFSs did not inhibit the growth of *S. aureus* indicator strain used in our study. These results are in agreement with previous studies, which show that the growth of 38 *Staphylococcus* spp. strains was not inhibited by crude bacteriocin of *E. faecium* EF9a, which possessed genes for enterocin A, B, P, and LB50 (Lauková et al., 2020). Antimicrobial metabolites produced by *E. faecalis* 58, *Enterococcus* sp. 226 and 423 strains are thermostable as they remained active after heating at 100°C for 20 min and lost activity after autoclaving (121°C, 15 min) only on 25%. This is in agreement with the results of other authors, who also reported about enterocins, that withstood heat treatment at 100°C and autoclaving (Kumar et al., 2010). At the same time, resistance to autoclaving may vary among described enterocins, some of them lost activity after autoclaving at 121°C (Alvarado et al., 2005). The loss of antibacterial activity of CFS after treatment with proteinase K, α-chymotrypsin, and trypsin was observed. Lipase and α-amylase did not affect the antimicrobial activity of CFS, indicating that the antimicrobial metabolites are proteinaceous in nature and do not depend on the lipid or carbohydrate moiety. These results agreed with previously reported data concerning enzymatic treatment sensitivity of studied enterocins (Du Toit et al., 2000; Cocolin et al., 2007; Al-Seraih et al., 2017), so we can assume that these strains are potential producers of bacteriocin-like substances (BLIS). We did not determine the effect of pH on CFS antagonistic activity in this study. However, we can assume that the antimicrobial metabolites are active at least in the pH range of 3.7–6.5 since there was no difference in the activity of the CFSs before and after neutralization. *Enterococcus* sp. 423, 226, and *E. faecalis* 58 strains were insensitive to their own CFS. Resistance in the bacteriocin-producing cells may indicate the expression of immunity peptides along with bacteriocin (Bastos et al., 2015; Teso-Pérez et al., 2021). Cross-resistance in the *E. faecalis* 58, *Enterococcus* sp. 423, and 226 strains may indicate a strong similarity of the BLIS produced by these strains.

No anti-listerial activity was observed only during the growth of *ent*-genes possessing *Enterococcus* sp. 423, 226, and *E. faecalis* 58 strains in 2% molasses, although in some cases viable cell counts were compatible with other media used. The absence of antagonistic action upon growth in 2% molasses was presumably attributable to the insufficiency of nutrients for enterocin production. This result is in agreement with other studies, that reported the absence of enterocins production when *Enterococcus* strains were grown in molasses (Todorov and Dicks, 2005; Coetzee et al., 2007). Similar to this result, authors have shown that *E. faecium* strains grew in cheese whey with no enterocin A production and produced maximum bacteriocin in cheese whey with supplemented of yeast extract (Mirhosseini and Emtiazi, 2011). At the same time, selected *Enterococcus* strains showed anti-listerial activity during growth in the medium that contains only CSL, peptone, and yeast extract. Although the сorn steep liquor is mainly used as a source of nitrogen, it also contains carbohydrates and other nutrients, in an amount sufficient for the growth and synthesis of bacteriocins (Coetzee et al., 2007). Thus, the production at least of enterocin P by strains *E. faecalis* 58, *Enterococcus* sp. 423, and *Enterococcus* sp. 226 when growing in four of five media used may be confirmed since the anti-listerial activity was detected in their cell-free supernatants. Furthermore, anti-enterococcal activity also was detected, CFSs inhibited the growth of *E. faecalis* indicator strains, but not *E. faecium* and *E. durans* strains. At the same time, enterocins B3A-B3B and B20A-B20B produced by *E. faecalis* strains were not active against the *E. faecalis* ATCC 29212, as was shown by authors (Al-Seraih et al., 2017).

Strains *E. faecalis* 888 and *E. durans* 248 possess only the *entA* gene and have anti-listerial activity only when grown in Medium II. Therefore, the *entA* gene in these strains was expressed only when growing on this medium. It is also possible that the expression also occurred in other media but in insufficient quantity for detection by well diffusion assay. As was shown by authors, *E. faecium* strains that possessed only the *entA* gene showed remarkably weaker anti-listerial action compared with isolates that possessed *entA-entB-entP* or *entA-entB* genes (Vandera et al., 2018). Strain *E. faecalis* 888 was susceptible to CFSs of *Enterococcus* sp. 423, 226, and *E. faecalis* 58, but strain *E. durance* 248 was not. Therefore, we cannot rule out the possibility that the antilisterial activity could be mediated by other bacteriocins, whose genes were not determined in this study. Furthermore, anti-listerial activity was detected in *E. faecalis* 220 and 266 strains when growing in MRS broth, but *entA*, *entB*, or *entP* genes were not detected in these strains. As was shown by the authors, *E. lactis* strains that harboured the *entP* gene, not showed antibacterial activity against *L. monocytogenes* (Morandi et al., 2013). Thus, the results of our study once again corroborate the data of other authors, that the medium composition has a great influence on LAB bacteriocin production (Audisio et al., 2001), and the most effective approach is to use both microbiological methods and screening for the presence of enterocin genes using PCR analysis.

It should be noted, that no antibacterial activity was observed against *P. aeruginosa*, *E. coli*, and *S. aureus* after centrifugation and removal of cells from bacterial cultures. The loss of antilisterial activity by filter-sterilized supernatants of *Enterococcus* strains the authors explained by the removal of all viable cells from broth supernatants (Vandera et al., 2019). The same observation about the need for cell-to-cell contact between the producer strain and the indicator strain was reported by other authors (Saraoui et al., 2016). This fact is of interest and could be attributed to the presence of other antimicrobial metabolites strongly associated with the bacterial cell surface. This may also be evidenced by smaller growth inhibition zones of these indicator strains compared to *L. monocytogenes* during initial screening. The levels of acid production (titrable acidity) were comparable in different strains (data not shown), so the antagonistic action is unlikely to be mediated by organic acid. The fact that some *Enterococcus* strains showed different spectra of activity when grown on five media may also indicate the production of specific antimicrobial metabolites. The strains *E. faecalis* 266 and 220 were active against *L. monocytogenes* when grown on MRS broth. At the same time, strain *E. faecalis* 266 showed activity only against *P. aeruginosa* on four other media, while strain *E. faecalis* 220 on the 2% molasses was active against *P. aeruginosa*, *E. coli*, and *S. aureus*. The absence of antibacterial activity in cell-free neutralized supernatants also may indicate the sensitivity of the antimicrobial metabolites to pH, as was shown by authors for some bacteriocins (Vandera et al., 2018). For example, the strong activity of Nisin at acidic pH is considerably reduced at a neutral pH range (Noutsopoulos et al., 2017). Thus, further research is needed to clarify this issue.

Although enterococci are widespread in fermented foods and used as a starter or protective cultures, some *Enterococcus* strains may harbor recognized virulence determinants and have caused nosocomial infections in humans (Ogier and Serror, 2008). Our previous preliminary study showed that autochthonous enterococci isolated from Ukrainian traditional dairy products can produce tyramine (95,4% of strains), 20% of strains exhibited β-hemolytic activity, and 56,76% were resistant to 5–11 antibiotics (Garmasheva et al., 2018). In particular, among seven potential bacteriocin producers selected in the present study, three strains (*Enterococcus* sp. 226, *Enterococcus* sp. 423, and *E. durans* 248) were β-hemolytic, and all strains were able to produce tyramine. Overall, seven *Enterococcus* strains exhibited susceptibility to 13 of 17 of the most clinically relevant antibiotics tested, including benzylpenicillin, vancomycin, and teicoplanin, with the exclusion of *E. faecalis* 266 and *E. faecalis* 220 that were resistant to benzylpenicillin and vancomycin, respectively. Further in-depth analysis of other putative virulence factors must be conducted to evaluate the potential for practical use of these bacteriocinogenic *Enterococcus* strains. The presence of virulence factors completely excludes the use of the bacteriocinogenic *Enterococcus* strains as a starter or protective culture for inoculation of raw materials or products, respectively. But such *Enterococcus* strains can be used as producers of bacteriocins followed by their isolation from the culture fluid and purification. It was shown, that CFS and semi-purified BLIS produced by *Enterococcus hirae* ST57ACC have no cytotoxic effect against HT-29 cells (Cavicchioli et al., 2019). Cell-free supernatant of bacteriocinogenic *E. faecium* TJUQ1 strain was used for the preparation of composite film with antibacterial activity (Feng et al., 2021). But the safety of each purified bacteriocin needs to be assessed before use in the food industry.

## 5. Conclusion

The present work is, to our knowledge, the first report on the screening of bacteriocinogenic *Enterococcus* strains isolated from Ukrainian traditional dairy products using low-cost by-product-based media. In this study, three *Enterococcus* strains that harbor genes of enterocins and produce BLIS with anti-listerial and anti-enterococcal activity were selected. We can assume that the use of the method described here will make it possible to exclude the stage of selecting and optimizing the medium for the large-scale production of bacteriocins. Strains *E. faecalis* 58, *Enterococcus* sp. 423, and *Enterococcus* sp. 226 are promising candidates for practical use as producers of bacteriocins with inhibitory activity against *L. monocytogenes* using molasses and steep corn liquor as cheap sources of carbon and nitrogen, that can significantly reduce the cost of industrial bacteriocin production. Further studies will be required to determine the dynamic of bacteriocin production, its structure, and mechanisms of antibacterial action.

## Data availability statement

The raw data supporting the conclusions of this article will be made available by the authors, without undue reservation.

## Author contributions

IG designed and performed the experiments, analyzed the results, and wrote the manuscript. LO performed the experiments. All authors contributed to the article and approved the submitted version.

## Conflict of interest

The authors declare that the research was conducted in the absence of any commercial or financial relationships that could be construed as a potential conflict of interest.

## Publisher’s note

All claims expressed in this article are solely those of the authors and do not necessarily represent those of their affiliated organizations, or those of the publisher, the editors and the reviewers. Any product that may be evaluated in this article, or claim that may be made by its manufacturer, is not guaranteed or endorsed by the publisher.
